# Resting Orientations of Dinosaur Scapulae and Forelimbs: A Numerical Analysis, with Implications for Reconstructions and Museum Mounts

**DOI:** 10.1371/journal.pone.0144036

**Published:** 2015-12-16

**Authors:** Phil Senter, James H. Robins

**Affiliations:** 1 Department of Biological Sciences, Fayetteville State University, Fayetteville, North Carolina, United States of America; 2 Department of Biology, Southeast Missouri State University, One University Plaza, Cape Girardeau, Missouri, 63701, United States of America; Perot Museum of Nature and Science, UNITED STATES

## Abstract

The inclination of the scapular blade and the resting pose of the forelimb in dinosaurs differ among reconstructions and among skeletal mounts. For most dinosaurian taxa, no attempt has previously been made to quantify the correct resting positions of these elements. Here, we used data from skeletons preserved in articulation to quantify the resting orientations of the scapula and forelimb in dinosaurs. Specimens were included in the study only if they were preserved lying on their sides; for each specimen the angle between forelimb bones at a given joint was included in the analysis only if the joint was preserved in articulation. Using correlation analyses of the angles between the long axis of the sacrum, the first dorsal centrum, and the scapular blade in theropods and *Eoraptor*, we found that vertebral hyperextension does not influence scapular orientation in saurischians. Among examined taxa, the long axis of the scapular blade was found to be most horizontal in bipedal saurischians, most vertical in basal ornithopods, and intermediate in hadrosauroids. We found that in bipedal dinosaurs other than theropods with semilunate carpals, the resting orientation of the elbow is close to a right angle and the resting orientation of the wrist is such that the hand exhibits only slight ulnar deviation from the antebrachium. In theropods with semilunate carpals the elbow and wrist are more flexed at rest, with the elbow at a strongly acute angle and with the wrist approximately at a right angle. The results of our study have important implications for correct orientations of bones in reconstructions and skeletal mounts. Here, we provide recommendations on bone orientations based on our results.

## Introduction

Interpretations of the resting positions of dinosaurian scapulae and forelimbs are inconsistent among museum mounts and illustrations. For a given taxon, the angle between the horizontal (the plane of the surface of the ground) and the long axis of the scapula differs among reconstructors, as does the resting pose of the forelimb in bipedal dinosaurs [1–3]. This is probably because before now there has been little information that is based on scientific research, to use as a basis for reconstructing forelimb resting poses in dinosaurs. Here, we seek to supply such information.

**Fig 1:**
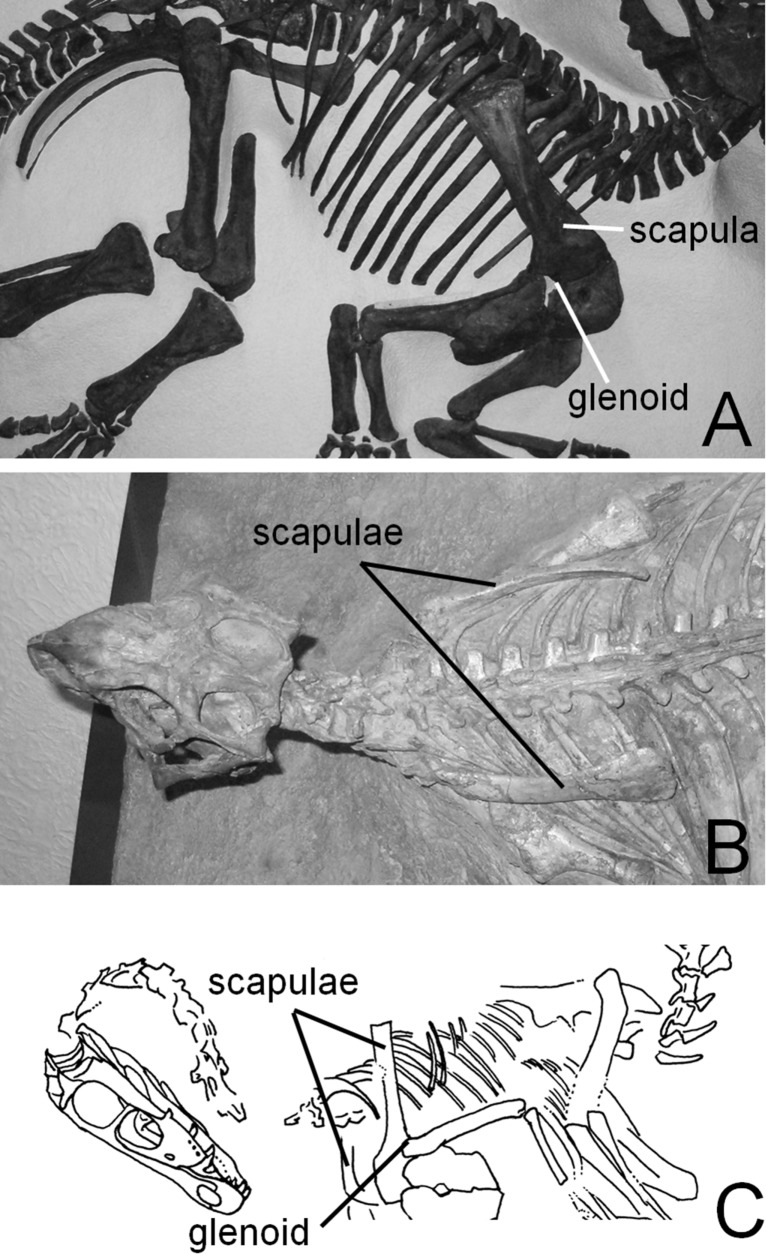
Dinosaur specimens preserved in articulation, showing that the glenoid is anteroventral to the ribcage and the scapular blades lie along the sides of the ribcage. A. CMN cast of AMNH 5351, *Centrosaurus apertus*. B. *Psittacosaurus mongoliensis*, AMNH 6254. C. *Velociraptor mongoliensis*, IGM 100/25, after reference [9]. See Table 1 for institutional abbreviations.

**Fig 2:**
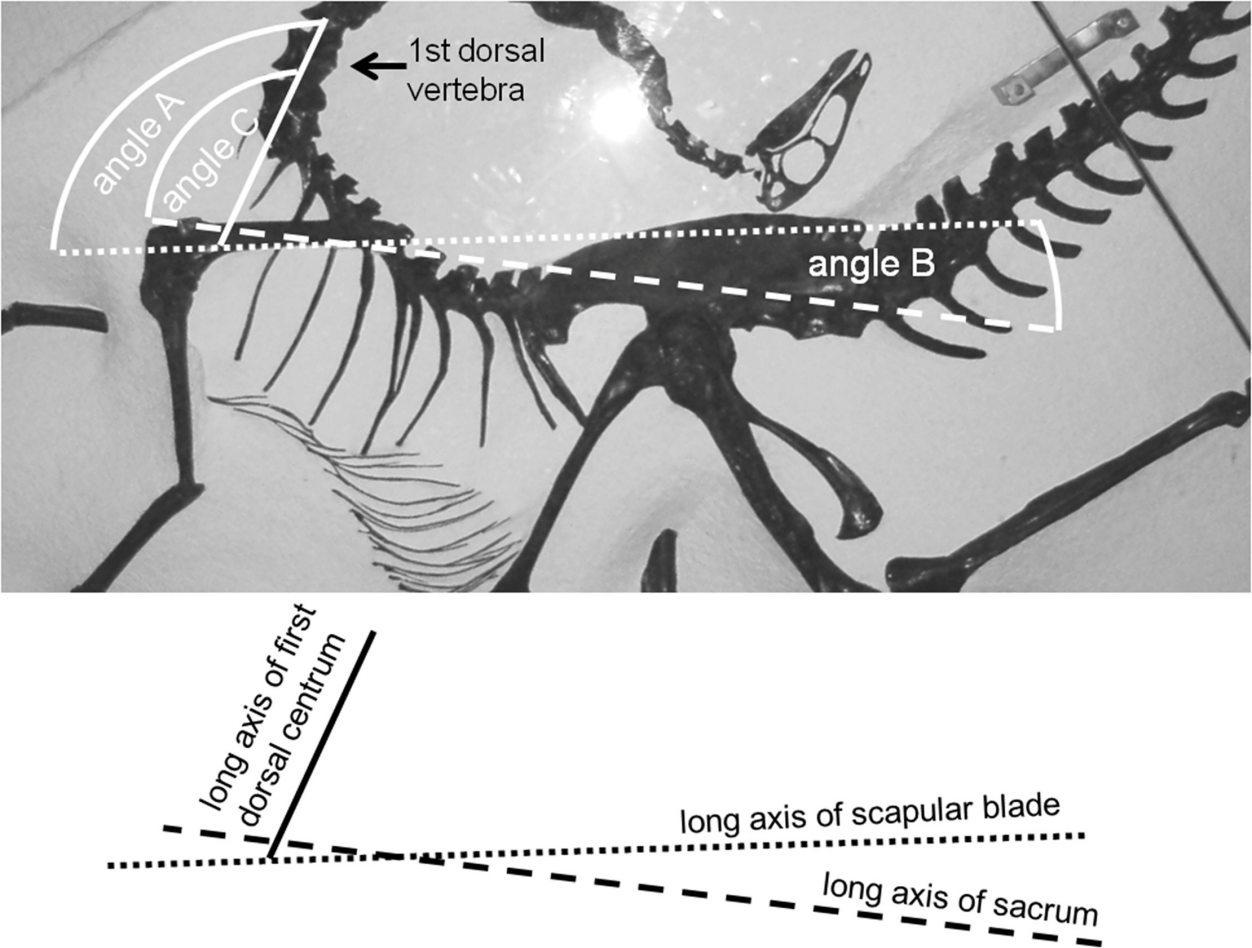
AMNH 5339, *Struthiomimus altus*, illustrating angles used in the study of resting scapular orientation. Solid straight line follows longitudinal axis of first dorsal vertebra, broken line with small dashes follows axis of scapular blade, and broken line with large dashes follows axis of sacrum. Angle A = angle between long axes of scapular blade and first dorsal centrum (116° in this case). Angle B = angle between long axes of scapular blade and sacrum (9° in this case). Angle C = angle between long axes of sacrum and first dorsal vertebra (107° in this case).

**Fig 3:**
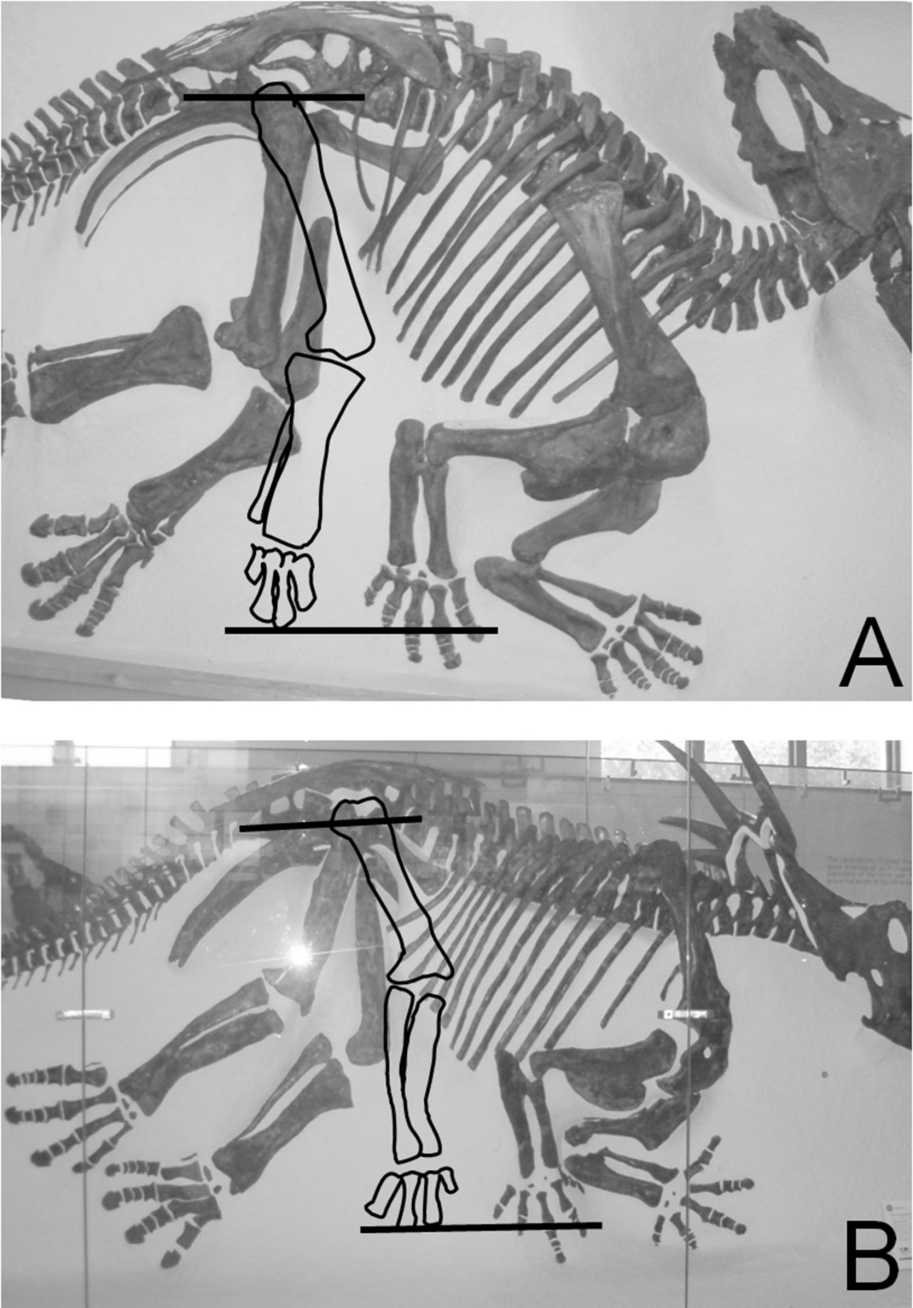
Ceratopsid skeletons preserved in articulation, showing that the ceratopsid sacrum is horizontal (parallel with the ground) when the humerus is held horizontally. The line through the long axis of the sacrum is subparallel with the line that serves as a proxy for the horizontal by connecting the tips of the metapodials of the right forelimb (with horizontal humerus) and an outline of those of the right hindlimb, with the latter rotated to simulate a normal standing pose. A. CMN cast of AMNH 5351, *Centrosaurus apertus*. B. AMNH 5372, *Styracosaurus albertensis*. See Table 1 for institutional abbreviations.

Dinosaur skeletons that are preserved in articulation can be used as a first estimate of the resting poses of scapulae and forelimbs. Such specimens show that the scapular blade is positioned along the sides of the ribcage with its flat internal surface subvertical and the glenoid facing ventrally (Figs 1, 2 and 3). This is true for both ornithischians and saurischians (Fig 1). Even in dromaeosaurid and oviraptorosaurian theropods, which are often depicted with the scapular blade dorsal to the ribcage with its flat internal surface subhorizontal as in extant birds [4–6], specimens preserved in articulation show that the scapulae are instead oriented as in other dinosaurs [7–12] (Fig 1C). Such is also the case with basal birds [12–14]. Skeletons that are preserved in articulation also reveal that the dinosaurian glenoid fossa is positioned as in extant non-avian tetrapods: immediately anteroventral to the first dorsal rib (Figs 1–3). However, that does not reveal the angle at which the long axis of the scapular blade was held with respect to the horizontal.

The pose of the bipedal saurischian forelimb has been investigated by several researchers [15–21]. Manual articulation of the humerus, radius, and ulna at the elbow so that opposing joint surfaces are connected reveals that the palms of bipedal saurischians faced medially [15–21]. However, that offers no clues regarding the angles at which the elbow and wrist were held in lateral view.

Here, we use data from specimens preserved in articulation to determine the resting poses of the scapular blade and forelimb in dinosaurs. Until now, most reconstruction of scapular and forelimb orientation in dinosaurs has been done without publication of reconstruction criteria. An exception is a previous study in which skeletons preserved in articulation were used to standardize theropod scapular orientation [17]. The previous study did not examine resting forelimb orientation and did not use statistical interpretation of numerical data to quantify the resting pose of the scapula. Numerical data were used in this way in one previous study on ceratopsians [22] and one on theropods [18], but this has not been done with other dinosaurian taxa. For most dinosaurian taxa, this study is the first to base scapular and forelimb orientation on statistical interpretation of numerical data.

## Materials and Methods

We ran three sets of analyses. First, we ran correlation analyses to determine whether vertebral hyperextension introduces artifacts into scapular orientation in saurischian dinosaurs. Next, we calculated mean scapular orientations in various saurischian and ornithischian dinosaur morphotypes. Afterwards, we calculated mean humeral, antebrachial, and metacarpal orientations in various saurischian and ornithischian dinosaur morphotypes.

For this study, we photographed specimens ourselves when possible. For specimens that we had not examined personally, we used published photographs. For such specimens, we included all that are represented by lateral-view photographs (or line drawings traced from such photographs) that fit the criteria below and that had been published by the end of 2014, to the best of our knowledge. For a given joint, a specimen was included in the sample only if the joint is preserved in articulation. A specimen was included only if the skeleton was preserved lying on its side, because all angles were measured in lateral view. Exceptions to this rule are AMNH 5060 (*Edmontosaurus annectens*) and MNA V 2623 (*Megapnosaurus kayentakatae*), which are preserved in three dimensions. Some skeletons that superficially appear to be preserved lying on their sides (e.g. AMNH 7223, *Coelophysis bauri*) were excluded from the sample, because vertebral orientation reveals that they were actually preserved ventral side down. Some skeletons preserved lying on their sides have one limb that is positioned high over the dorsum (e.g. HMN 1880/81, *Archaeopteryx lithographica*; IVPP V 13352, *Microraptor zhaoianus*); for such specimens, that limb was omitted from the sample, because it is taphonomically displaced. The other limb was included in the sample if it exhibits no signs of taphonomic displacement. The juvenile hadrosaurid “*Procheneosaurus praeceps*” (AMNH 5340), which is mounted in articulation, was omitted because museum records do not reveal whether or not it was found in articulation as mounted.

### Correlation analyses of vertebral hyperextension and scapular orientation

In bipedal saurischians the sacrum is held horizontally [21,23], which suggests that the angle between the scapular blade and the sacrum represents the resting orientation of the scapula relative to the ground. However, saurischian dinosaur skeletons are often preserved with the vertebral column hyperextended (curved so that it is concave dorsally), a problem absent in ornithopods and ceratopsids because a network of ossified tendons holds their dorsal vertebrae in place. The magnitude of the hyperextension differs among saurischian specimens. Before calculating scapular angle means, we therefore considered it necessary to determine whether vertebral hyperextension influenced scapular orientation.

We measured orientations for three scapular angles in bipedal saurischians (Fig 2): angle A (angle between long axis of distal scapular blade and longitudinal axis of first dorsal centrum), angle B (angle between long axis of distal scapular blade and longitudinal axis of sacrum), and angle C (angle between longitudinal axis of the sacrum and that of the first dorsal centrum; angle C = angle A–angle B) (Table 1). Specimens were included in the calculations only if their scapulae were not taphonomically displaced away from the pectoral region of the skeleton, and if the long axis of the sacrum could be discerned.

**Table 1:** Data used in correlation analyses that tested hypotheses of resting scapular orientation in bipedal saurischians. See Materials and Methods section for descriptions of angles A, B, and C. Asterisked scapulae exhibit a scapular tip that extends high above the vertebral column. For angles A and B, a negative number represents an orientation in which the tip of the scapular blade is further from the vertebral column than the acromion is (in most dinosaurs, the opposite is the case). Institutional abbreviations (for this and subsequent tables): AM = Amherst College Museum, Amherst, Massachusetts. AMNH = American Museum of Natural History, New York City, New York, United States. BHI = Black Hills Institute of Geological Research, Hill City, South Dakota, United States. BMMS = Bürgermeister Müller MuseumSolnhofen, Solnhofen, Germany. BSP = Bayerische Staatsammlung für Paläontologie und Historische Geologie, Munich, Germany. CAGS = China Academy of Geological Sciences, Beijing, China. CHG = Chengdu College of Geology, Sichuan Province, China. CM = Carnegie Museum of Natural History, Pittsburgh, Pennsylvania. CMN = Canadian Museum of Nature, Ottawa, Ontario, Canada. DINO = Dinosaur National Monument, Jensen, Utah, United States. HMN = Museum für Naturkunde, Berlin, Germany. IGM = Mongolian Institute of Geology, Ulaan Bator, Mongolia. IRSNB = Institut Royal des Sciences Naturelles de Belgique, Brussels, Belgium. IVPP = Institute of Vertebrate Paleontology and Paleoanthropology, Beijing, China. JM = Jura Museum, Eichstätt, Germany. LH = Long Hao Institute for Paleontology, Hohhot, Nei Mongol Autonomous Region, China. LH = Universidad Autónoma de Madrid, Madrid, Spain. LPM = Liaoning Paleontological Museum, Liaoning Province, China. MCF = Museo Carmen Funes, Plaza Huincul, Argentina. MCZ = Museum of Comparative Zoology, Cambridge, Massachusetts, United States. MNA = Museum of Northern Arizona, Flagstaff, Arizona, United States. MNHN = Muséum National d’Histoire Naturelle, Paris, France. MOR = Museum of the Rockies, Bozeman, Montana, United States. NGMC = National Geological Museum of China, Beijing, China. NIGP = Nanking Institute of Geology and Paleontology, Beijing, China. PVSJ = Museo de San Juan, San Juan, Argentina. ROM = Royal Ontario Museum, Toronto, Ontario, Canada. SAM = South African Museum, Cape Town, South Africa. SBA = Sopritendeza per i Beni Archeologici di Salerno, Avellino, Benevento e Caserta, Italy. SC = Italian state collections (no associated city). SMNS = Staatliches Museum für Naturkunde, Stuttgart, Germany. STM = Shandong Tianyu Museum of Nature, Pingyi, Shandong, China. TMP = Royal Tyrrell Museum of Palaeontology, Drumheller, Alberta, Canada. UCMZ = University of Calgary Museum of Zoology, Calgary, Alberta, Canada. ULR = Museo de Ciencias Naturales de la Universidad de La Rioja, La Rioja, Argentina. YFGP = Yizhou Fossil and Geology Park, Liaoning, China. YPM = Yale Peabody Museum, New Haven Connecticut, United States.

Species, specimen	Side	angle A	angle B	angle C	Image source
*Anchiornis* sp., IVPP V 16055	r	37°	13°	24°	photo by P. S.
*Archaeopteryx lithographica*, JM 2257	l	88°	25°	63°	fig 3 of reference [24]
	r	89°	26°	63°	
*Archaeopteryx lithographica*, HMN 1880/81	l	37°	5°	32°	photo of cast by P. S.
	r	42°	10°	32°	
*Aurornis xui*, YFGP T-5198	r	26°	26°	0°	fig 1 of reference [25]
*Caudipteryx* sp., IVPP V 12430	l	45°	28°	17°	pl 2, 5, 7 of reference [8]
	r*	52°	35°	17°	
*Caudipteryx zoui*, BPM 0001	l*	58°	21°	37°	pl 1, 4 of reference [8]
	r	42°	5°	37°	
*Compsognathus longipes*, BSP 1563	l*	57°	0°	57°	fig 1 of reference [26]
*Compsognathus longipes*, MNHN CNJ 79	l	129°	22°	107°	fig 3 of reference [27]
*Eoraptor lunensis*, PVSJ 512	l	12°	63°	-51°	photo of cast by P. S.
*Eosinopteryx brevipenna*, YFGP-T5197	l	38°	0°	38°	fig 1 of reference [14]
	r	41°	9°	38°	
*Gorgosaurus libratus*, AMNH 5428	r	42°	-17°	59°	fig 1 of reference [28]
*Huaxiagnathus orientalis*, CAGS IG 02–301	r	22°	21°	1°	fig 1 of reference [29]
*Microraptor gui*, IVPP V 13352	l	20°	20°	0°	fig 1 of reference [30]
	r*	7°	7°	0°	
*Oviraptor philoceratops*, AMNH 6517	l	7°	-	-	p 660 of reference [2]
*Scipionyx samniticus*, SBA-SA 163760	r*	60°	51°	11°	fig 1, 5 of reference [31]
*Sciurumimus albersdoerferi*, BMMS BK 11	l	30°	17°	13°	fig 1 of reference [32]
*Similicaudipteryx* sp., STM 4–1	r	-	55°	-	fig 1a of reference [33]
*Sinornithomimus dongi*, IVPP V 1197–4	r	19°	22°	-3°	fig 1 of reference [34]
*Sinosauropteryx prima*, NIGP 127586	r	49°	35°	14°	front cover of reference [35]
*Sinosauropteryx prima*, NIGP 127587	l*	67°	34°	33°	fig 1, 8 of reference [36]
	r	41°	8°	33°	
*Struthiomimus altus*, AMNH 5339	l	116°	9°	107°	pl 24 of reference [37]
*Velociraptor mongoliensis*, IGM 100/25	l*	40°	77°	-37°	p 24–25 of reference [9]
*Wellnhoferia grandis*, holotype	r	-	-6°	-	fig 1 of reference [38]

The basal birds *Archaeopteryx*, *Wellnhoferia*, and *Aurornis* are included in the bipedal saurischian sample because their scapulae are configured as in non-avian theropods. That is, their scapulae are along the sides of the ribcage, with the glenoid facing ventrally [12,13,25], unlike extant birds, in which the scapula is dorsal to the ribcage and the glenoid faces laterally. Disagreement as to whether the one known specimen of *Wellnhoferia* should be referred to *Archaeopteryx* [13,38] does not affect the specimen’s usefulness to this study, because taxonomic assessment does not change preserved scapular orientation.

We ran correlation analyses of angle A versus angle C, for angle B versus angle C, and for angle A versus angle C in bipedal saurischians. We used only one scapula (the left one where possible) from each specimen in the correlation analysis to avoid artifacts resulting from non-independence of scapular angles within a specimen. The sample size was 27. We used angle C to quantify the magnitude of vertebral hyperextension. Because all three correlations involve the same data, it was necessary to use a stringent alpha level for our statistics in order to avoid possible inflation of type 1 error rates. We therefore used a Bonferroni adjustment of 0.05/3 = 0.017 as our adjusted alpha level. The correlation analyses tested four hypotheses:

*Hypothesis 1*: *As the vertebral column hyperextends*, *the scapula changes its orientation relative to the sacrum*. In other words, as angle C changes, angle B changes. This hypothesis predicts that angles B and C are correlated.

*Hypothesis 2: As the vertebral column hyperextends, the scapula changes its orientation relative to the first dorsal vertebra*. In other words, as angle C changes, angle A also changes. This hypothesis predicts that angles A and C are correlated.

*Hypothesis 3*: *As the vertebral column hyperextends*, *scapular orientation relative to the sacrum remains constant*. In other words, as angle C increases, angle B remains constant, which means that angle A increases. This hypothesis predicts that angles A and C are correlated. It also predicts that angles C and A have a direct relationship: as one increases, so does the other. It further predicts that angle B is not correlated with angle A or C, because angle B is constant.

*Hypothesis 4: As the vertebral column hyperextends, scapular orientation relative to the first dorsal vertebra remains constant*. In other words, as angle C increases, angle B decreases. This hypothesis predicts that angles B and C are correlated and have an inverse relationship. It further predicts that angle A is not correlated with angle B or C, because angle A is constant.

In many dinosaur specimens that are preserved in articulation, the tip of the scapula protrudes high above the vertebral column (Table 1). Such protrusion indicates taphonomic displacement. These specimens were included in the sample used in the test of Hypotheses 1–4, because they were deemed potentially informative as to the relationship between scapular angles and vertebral hyperextension.

The results of the correlation analyses that tested Hypotheses 1–4 are as follows. Angles A and B are not significantly correlated (r = -3.256, P > 0.05). Angles A and C are significantly correlated (r = 3.695, P < 0.01) and have a direct relationship. Angles B and C are not significantly correlated (r = -0.601, P > 0.05). These results support Hypotheses 2 and 3and falsify Hypotheses 1 and 4. Therefore, during vertebral hyperextension the scapula maintains its orientation relative to the sacrum. We therefore chose angle B as our measure of the resting orientation of the scapula in bipedal saurischians, for our subsequent calculations of mean scapular orientations.

In a previous attempt to standardize scapular orientation in dinosaurs, an equivalent of angle A was used as a proxy for the inclination of the scapular blade relative to the horizontal [17]. However, here we find that angle A varies according to the magnitude of vertebral hyperextension. Because angle B does not, it is a better proxy for the inclination of the scapular blade relative to the horizontal.

### Calculation of mean scapular orientations

No single species of dinosaur is represented by a large enough sample size of skeletons preserved in articulation to calculate a reliable mean scapular orientation for the species. Therefore, we collected data from a wide spectrum of dinosaur taxa (Tables 1–3) and divided them into four scapular-orientation morphotypes: theropods + *Eoraptor*; ceratopsids; basal ornithischians and basal (non-hadrosauroid) ornithopods; and hadrosauroids. We then calculated the mean scapular orientation for each morphotype. We used rectangular coordinates of these angles to compute each mean, because use of arithmetic values for angles can generate nonsensical means [39]. Specimens were included in these calculations only if their scapulae were preserved in a reasonable state of articulation with the rest of the skeleton, and if the long axis of the sacrum could be discerned. Specimens with the tip of the scapula protruding high above the vertebral column (Table 1) were omitted from these calculations, because their scapulae are taphonomically displaced.

**Table 2:** Data used to calculate mean resting orientations of ornithischian scapular angles in lateral view. See Materials and Methods section for description of angle B. Group means shown without confidence intervals are those for which sample size is too small to derive 95% confidence intervals (n < 8). See Table 1 for institutional abbreviations.

Species, specimen	Side	angle B	Image source
**Ceratopsids**			
*Anchiceratops longirostris*, CMN FV 8535	r	71°	pl 10 of reference [40]
*Centrosaurus nasicornis*, AMNH 5351	r	41°	pl 11 of reference [41]
*Styracosaurus albertensis*, AMNH 5376	l	41°	fig 4, 5 of reference [42]
*Triceratops horridus*, BHI 126406	r	65°	p 204 of reference [43]
**Basal ornithopods and basal ornithischians**			
*Heterodontosaurus tucki*, SAM K 1332	l	75°	photo of cast by P. S.
*Othnielia rex*, MCZ 4454	r	72°	pl 4 of reference [44]
*Thescelosaurus neglectus*, ROM 8537	r	64°	fig 17 of reference [45]
**Hadrosauroids**			
*Corythosaurus casuarius*, AMNH 5240	l	32°	fig 13, 14 of reference [46]
	r	46°	
*Edmontosaurus annectens*, CMN FV 8399	l	76°	photo by P. S.
*Kritosaurus incurvimanus*, ROM 764	r	73°	pl 1 of reference [47]
*Parasaurolophus walkeri*, ROM 768	l	22°	pl 1 of reference [48]
*Saurolophus osborni*, AMNH 5220	r	35°	p 141 of reference [49]
*Tethyshadros insularis*, SC 57021	r	52°	fig 1 of reference [50]

**Table 3:** Data used to calculate mean resting orientations of dinosaurian forelimb joints in lateral view. l = left, r = right, S = shoulder angle, E = elbow angle, W = wrist angle. See Materials and Methods section for descriptions of angles. See Table 1 for institutional abbreviations.

Species, specimen	Side	S	E	W	Image Source
**Theropods without semilunate carpal**					
*Allosaurus fragilis*, DINO 11541	l	-	-	85°	fig 1 of reference [51]
*Compsognathus longipes*, BSP ASI 563	l	83°	88°	-	fig 1 of reference [26]
*Compsognathus longipes*, MNHN CNJ 79	l	87°	104°	-	fig 9 of reference [27]
	r	75°	147°	146°	
*Guanlong wucaii*, IVPP V 14532	r	39°	70°	135°	fig 1 of reference [52]
*Huaxiagnathus orientalis*, CAGS IG 02–301	l	-	-	170°	fig 1 of reference [29]
	r	49°	110°	159°	
*Megapnosaurus kayentakatae*, MNA V 2623	l	8°	-	-	fig 1 of reference [53]
*Ornithomimus edmontonicus*, CMN FV 8632	r	-	157°	153°	fig 3 of reference [54]
*Pelecanimimus polyodon*, LH 7777	l	-	-	178°	photos by P. S.
	r	-	-	175°	
*Scipionyx samniticus*, SBA-SA 163760	l	-	85°	150°	fig 1, 5 of reference [31]
	r	59°	37°	152°	
*Sciurumimus albersdoerferi*, BMMS BK 11	l	35°	55°	24°	fig 1 of reference [32]
*Sinornithomimus dongi*, IVPP V 11797–4	r	12°	32°	150°	fig 1 of reference [34]
*Sinornithomimus dongi*, IVPP V 11797–18	l	-	-	174°	fig 16 of reference [55]
*Sinornithomimus dongi*, LH PV 6	l	112°	118°	-	fig 3 of reference [56]
*Sinosauropteryx prima*, NIGP 127586	l	-	101°	-	front cover of reference [35]
	r	-	96°	-	
*Sinosauropteryx prima*, NIGP 127587	l	-	89°	154°	fig 1, 8 of reference [36]
	r	86°	93°	145°	
*Struthiomimus altus*, AMNH 5339	l	89°	134°	167°	pl 24 of reference [37]
*Struthiomimus altus*, UCMZ(VP) 1980.1	l	48°	72°	156°	fig 1 of reference [57]
*Struthiomimus* sp., BHM 1266 (cast)	l	-	89°	172°	photo by P. S.
**Theropods with semilunate carpal**					
*Anchiornis* sp., IVPP V 16055	r	45°	51°	126°	photo by P. S.
*Archaeopteryx lithographica*, HMN 1880/81	r	38°	37°	115°	photo of cast by P. S.
*Archaeopteryx lithographica*, JM 2257	r	49°	37°	111°	fig 3 of reference [24]
*Aurornis xui*, YFGP T-5198	r	40°	55°	117°	fig 1 of reference [25]
*Caudipteryx dongi*, IVPP V 12344	l	-	92°	150°	pl 2 of reference [7]
	r	-	95°	135°	
*Caudipteryx zoui*, NGMC 97-4-A	l	-	-	147°	photo by P. S.
	r	68°	82°	-	
*Caudipteryx zoui*, BPM 0001	l	-	126°	120°	pl 1, 4 of reference [8]
	r	70°	110°	145°	
*Caudipteryx* sp., IVPP V 12430	l	61°	77°	49°	pl 2, 5, 7 of reference [8]
*Cryptovolans pauli*, LPM 0200	l	52°	46°	63°	fig 1 of reference [58]
*Eosinopteryx brevipenna*, YFGP-T5197	l	42°	41°	121°	fig 1 of reference [14]
	r	60°	51°	128°	
*Jinfengopteryx elegans*, CAGS-IG-04-0801	r	32°	59°	126°	fig 1 of reference [10]
*Khaan mckennai*, IGM 100/1002	r	100°	60°	48°	photo by P. S.
*Khaan mckennai*, IGM 100/1127	r	-	30°	81°	photo by P. S.
*Microraptor gui*, IVPP V 13352	l	56°	58°	92°	fig 1 of reference [30]
	r	-	40°	67°	
*Microraptor gui*, IVPP V 17972A	r	-	41°	107°	fig 1 of reference [59]
*Oviraptor philoceratops*, AMNH 6517	l	72°	40°	63°	p 660 of reference [2]
*Similicaudipteryx* sp., STM 4–1	r	-	50°	145°	fig 1a of reference [33]
*Velociraptor mongoliensis*, IGM 100/982	l	30°	-	-	fig 27 of reference [60]
*Wellnhoferia grandis*, holotype	r	22°	20°	90°	fig 1 of reference [38]
**Basal sauropodomorphs**					
*Anchisaurus polyzelus*, AM 4/109	r	-	-	188°	fig 7 of reference [61]
*Ammosaurus major*, YPM 209	r	-	51°	-	fig 30 of reference [61]
*Eoraptor lunensis*, PVSJ 512 (cast)	l	-	87°	155°	photo of cast by P. S.
*Plateosaurus engelhardti*, SMNS F61	r	-	-	140°	photo by M. F. Bonnan
*Riojasaurus incertus*, ULR 56	l	-	-	178°	fig 2 of reference [62]
**Bipedal ornithischians**					
*Agilisaurus multidens*, CHG T6001	l	-	71°	-	p 92 of reference [2]
	r	112°	140°	147°	
*Edmontosaurus annectens*, CMN FV 8399	l	107°	180°	142°	photo by P. S.
	r	-	148°	132°	
*Heterodontosaurus tucki*, SAM K 1332	l	73°	27°	-	photo of cast by P. S.
	r	-	-	178°	
*Kritosaurus incurvimanus*, ROM 764	l	-	117°	194°	pl 1 of reference [47]
	r	128°	139°	191°	
*Othnielia rex*, MCZ 4454	l	-	150°	-	pl 4 of reference [44]
	r	91°	108°	-	
*Parasaurolophus walkeri*, ROM 768	l	67°	131°	180°	pl 1 of reference [48]
	r	-	130°	180°	
*Tethyshadros insularis*, SC 57021	r	80°	129°	155°	fig 1 of reference [50]
*Thescelosaurus neglectus*, USNM 7757	l	-	120°	146°	fig 11 of reference [63]
*Thescelosaurus neglectus*, ROM 8537	l	-	130°	168°	fig 17 of reference [45]
	r	60°	63°	172°	

In ornithopods the posterior vertebral column is subhorizontal [64]. This allows the long axis of the sacrum to be used as a reasonable proxy for the horizontal. We therefore chose Angle B as our measure of the resting orientation of the scapula in ornithopods.

Use of the sacrum as a proxy for the horizontal is controversial in ceratopsids, because some previous authors have reconstructed ceratopsids with strongly slanted sacra [65]. However, vertebral anatomy suggests that the ceratopsid sacrum was horizontal [66], and specimens preserved in articulation reveal that the long axis of the sacrum is parallel with the horizontal when the limbs are oriented in a standing pose [22] (Fig 3). As shown in Fig 3, this interpretation depends on a horizontal orientation of the humerus, and researchers generally agree that the ceratopsid humerus was held horizontally, even though they disagree about how far laterally the elbows were everted [67–70]. Manipulations by P. S. of NMC 344 (*Styracosaurus albertensis*) confirm that the posterior location of the humeral head keeps the humerus subhorizontal when it is pressed into the glenoid in Ceratopsidae. Incidentally, manipulations of casts confirm that such is the case also with basal ceratopsians [22]. Because the sacrum was subhorizontal in ceratopsids, we chose angle B as our measure of the resting orientation of the scapula in ceratopsids.

To determine whether scapular orientation differs between dinosaurian groups, it would have been ideal to use the Watson-Williams two-sample test for differences between mean angles [39]. However, our sample sizes for some groups are too small for this, so we used a different method. For the mean of each measurement in each dinosaurian group we used formulas in reference [39] to compute 95% confidence intervals where permitted by large enough sample sizes (n ≥ 8) (Table 4). We considered orientations different between groups if the confidence intervals do not overlap. If the confidence intervals overlap we considered orientations not to be demonstrably different between groups. Although use of confidence intervals in this way can yield misinterpretation [71], it the best approach possible with sample sizes as low as those used here.

**Table 4:** Mean resting orientations of dinosaurian scapulae in lateral view, with 95% confidence intervals (L_1_ and L_2_) for the mean of the one group with a large enough sample size to calculate confidence intervals, and with *n* in parentheses. See Materials and Methods section for description of angle B.

Group	Angle B
Bipedal saurischians	21° (27); L_1_ = 15°; L_2_ = 27°
Ceratopsids	55° (4)
Basal ornithopods and basal ornithischians	70° (3)
Hadrosauroids	48° (7)

### Calculation of mean forelimb bone orientations

We define the resting pose of the forelimb as the orientation of bones at the shoulder, elbow, and wrist when all muscles of the forelimb are relaxed. In the resting pose, elastic recoil of soft tissues causes considerable shoulder retraction, elbow flexion, and wrist abduction in extant tetrapods, as we have personally observed. At death, muscles relax, and unobstructed forelimbs are drawn into the resting pose by elastic recoil, as we have personally observed in reptiles and birds. Unobstructed forelimbs of dead dinosaurs ought therefore to exhibit the resting pose.

No single species of dinosaur is represented by a large enough sample size of skeletons to calculate a reliable mean joint orientation for the species. Therefore, we collected data from a wide spectrum of bipedal dinosaurs (Table 3) and divided them into four forelimb morphotypes: theropods without carpals of semilunate shape; theropods with semilunate distal carpals; basal sauropodomorphs; and bipedal ornithischians (ornithopods and basal ornithischians). For each morphotype we calculated the mean orientation for the shoulder, elbow, and wrist. Because specimens of Caudipteridae (*Caudipteryx* and *Similicaudipteryx*) exhibit very different forelimb angles from other theropods with semilunate carpals, calculations were undertaken separately for Caudipteridae.

**Fig 4:**
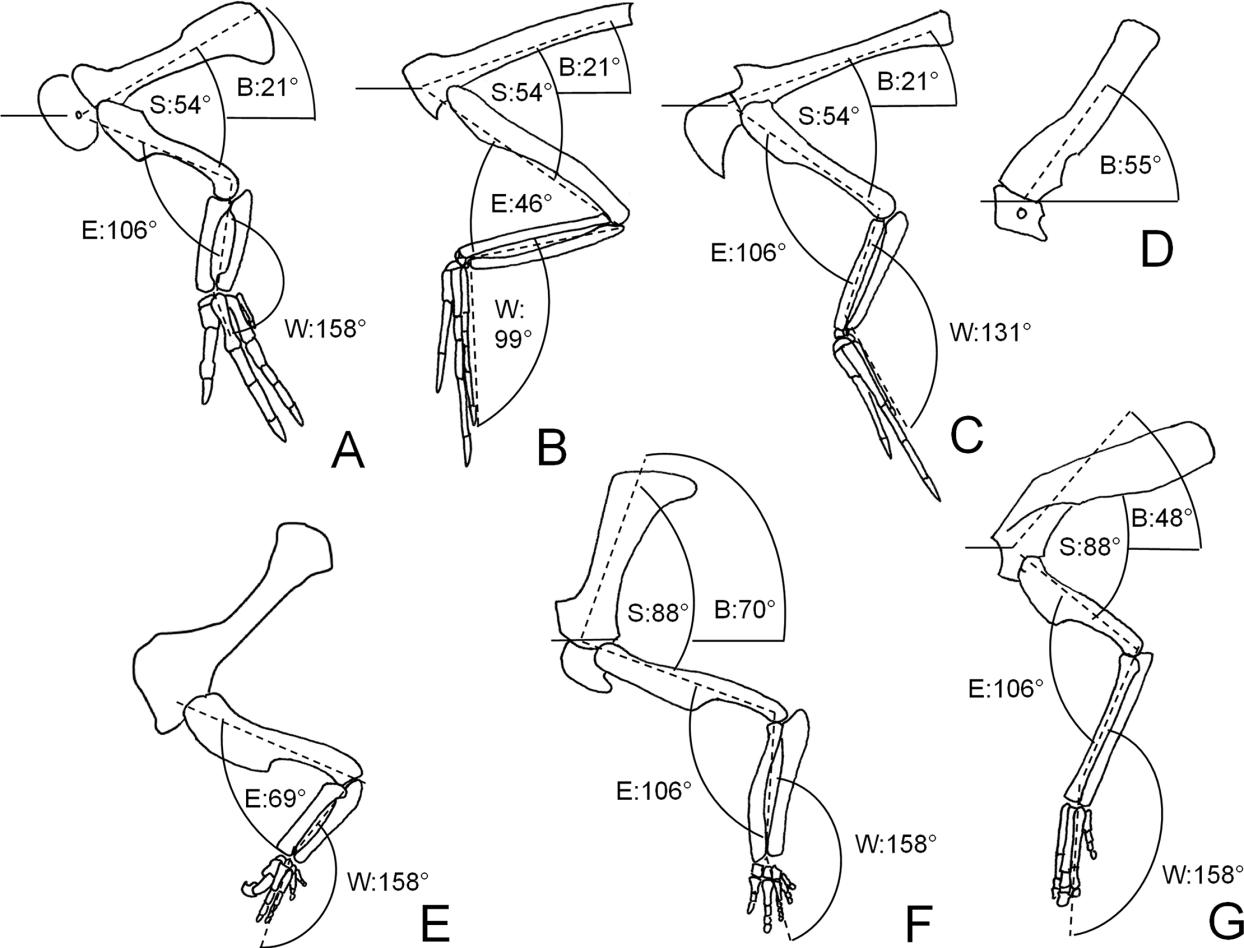
Pectoral girdles and forelimbs of dinosaurs in left lateral view, depicting resting scapular and forelimb orientations in different dinosaurian groups as recommended according to the results of this study. In each case, solid horizontal line is parallel to long axis of sacrum. A. theropods without semilunate carpals (*Dilophosaurus wetherilli*, UCMP 37302). B. theropods with semilunate carpals other than *Caudipteryx* (*Velociraptor mongoliensis*, after reference [60]). C. *Caudipteryx* (*C*. sp., after reference [8]). D. ceratopsids (*Styracosaurus albertensis*, NMC 344). E. basal sauropodomorphs (*Plateosaurus engelhardti*, AMNH 6810). F. non-hadrosaurian ornithopods (*Thescelosaurus neglectus*, reference [62]). G. hadrosaurids (*Parasaurolophus walkeri*, after reference [48]). Angle labels: B = scapular orientation relative to long axis of sacrum. E = elbow angle. S = shoulder angle. W = wrist angle. See Materials and Methods section for descriptions of angles.

For each specimen, the shoulder angle (Table 3: angle S) was measured as the angle between the long axis of the proximal part of the scapular blade and a tangent to the humeral midshaft in lateral view. The elbow angle (Table 3: angle E) was measured as the angle between a tangent to the humeral midshaft and a line connecting the centers of the proximal and distal extremities of the radius. The wrist angle (Table 3: angle W) was measured as the angle between a line connecting the tip of the olecranon process to the center of the distal extremity of the ulna, and a line connecting the centers of the proximal and distal extremities of the second metacarpal. We used the mean shoulder angle, the mean elbow angle, and the mean wrist angle of the sample of each morphotype to estimate the resting forelimb pose of the morphotype (Tables 5 and 6) (Fig 4). As in the calculations for group means of scapular angle B, we used rectangular coordinates of the angles to compute the means [39]. As with scapular orientations, we used a lack of overlap between 95% confidence intervals around mean angles to determine whether joint orientations differ between morphotypes. For any two or more groups for which the orientation was found not to differ at a given joint, we calculated a combined group mean and its confidence intervals (Table 4).

**Table 5:** Mean resting orientations of dinosaurian forelimb joints in lateral view, with 95% confidence intervals (L_1_ and L_2_) of the means of the groups and combined groups with large enough sample sizes, and with *n* in parentheses. S = shoulder angle, E = elbow angle, W = wrist angle. See Materials and Methods section for descriptions of angles.

Group	S	E	W
Theropods without semilunate carpal	57° (12); L_1_ = 44°; L_2_ = 70°	90° (16); L_1_ = 79°; L_2_ = 101°	150° (15); L_1_ = 140°; L_2_ = 160°
Theropods with semilunate carpal (other than Caudipteridae)	49° (13); L_1_ = 40°; L_2_ = 58°	46° (14); L_1_ = 43°; L_2_ = 49°	99° (14); L_1_ = 90°; L_2_ = 108°
Caudipteridae	66° (3)	90° (7)	131° (7)
Basal sauropodomorphs	- (0)	69° (1)	169° (3)
Bipedal ornithischians	88° (8); L_1_ = 75°; L_2_ = 102°	100° (15)164° (12); L_1_ = 93°; L_2_ = 107°	88° (8); L_1_ = 159°; L_2_ = 169°
**Combined groups**			
All theropods	54° (28); L_1_ = 47°; L_2_ = 61°	73° (38); L_1_ = 66°; L_2_ = 80°	126° (36); L_1_ = 119°; L_2_ = 133°
Theropods without semilunate carpals + bipedal ornithischians	71° (20); L_1_ = 62°; L_2_ = 80°	106° (31); L_1_ = 98°; L_2_ = 114°	157° (27); L_1_ = 150°; L_2_ = 164°
Theropods without semilunate carpals + basal sauropodomorphs + bipedal ornithischians	71° (20); L_1_ = 80°; L_2_ = 62°	77° (33); L_1_ = 70°; L_2_ = 84°	158° (31); L_1_ = 152°; L_2_ = 164°

**Table 6:** Recommended orientations of dinosaurian scapulae and forelimb joints in lateral view, for use in reconstructions and skeletal mounts, based on results of this study. See Materials and Methods for descriptions of each angle.

Group	Angle B	Shoulder	Elbow	Wrist
Theropods without semilunate carpal	21°	54°	106°	158°
Theropods with semilunate carpal, except Caudipteridae	21°	54°	46°	99°
Caudipteridae	21°	54°	106°	131°
Basal sauropodomorphs	-	-	69°	158°
Ceratopsids	55°	-	-	-
Basal ornithopods and basal ornithischians	70°	88°	106°	158°
Hadrosauroids	48°	88°	106°	158°

## Results

Group means and combined group means for all measurements are given in Tables 4 and 5, with 95% confidence intervals for the means of the groups and combined groups with large enough samples. Group means for scapular inclination (angle B) differ among all groups, and the confidence intervals of the one group with a large enough sample size to calculate them (bipedal sauriachians) do not overlap the group mean of any other group. Therefore, according to our method, scapular orientation differs among all groups.

For the shoulder angle, confidence intervals overlap between all theropod groups. Therefore, according to our method, the shoulder angle is not demonstrably different between theropod groups. The confidence intervals do not overlap between theropods and bipedal ornithischians. Therefore, according to our method, the shoulder angle differs between theropods and bipedal ornithischians.

For the elbow angle, confidence intervals overlap between theropods without semilunate carpals, Caudipteridae, and bipedal ornithischians. Therefore, according to our method, the elbow angle is not demonstrably different between those three groups. The mean elbow angle of basal sauropodomorphs does not overlap the confidence intervals of any other group. The confidence intervals of the elbow angle for theropods with semilunate carpals do not overlap the confidence intervals of the other groups or the mean for sauropodomorphs. Therefore, according to our method, the elbow angle differs between basal sauropodomorphs, theropods with semilunate carpals, and a combination of the other groups.

For the wrist angle, confidence intervals overlap between theropods without semilunate carpals, basal sauropodomorphs, and bipedal ornithischians. Therefore, according to our method, this angle is not demonstrably different between those groups. The mean wrist angle in Caudipteridae is not within the confidence intervals of the other groups. Confidence intervals for the wrist angle differ between theropods with semilunate carpals (without Caudipteridae) and all other groups. Therefore, according to our method, the wrist angle differs between Caudipteridae, other theropods with semilunate carpals, and a combination of the other groups.

**Fig 5:**
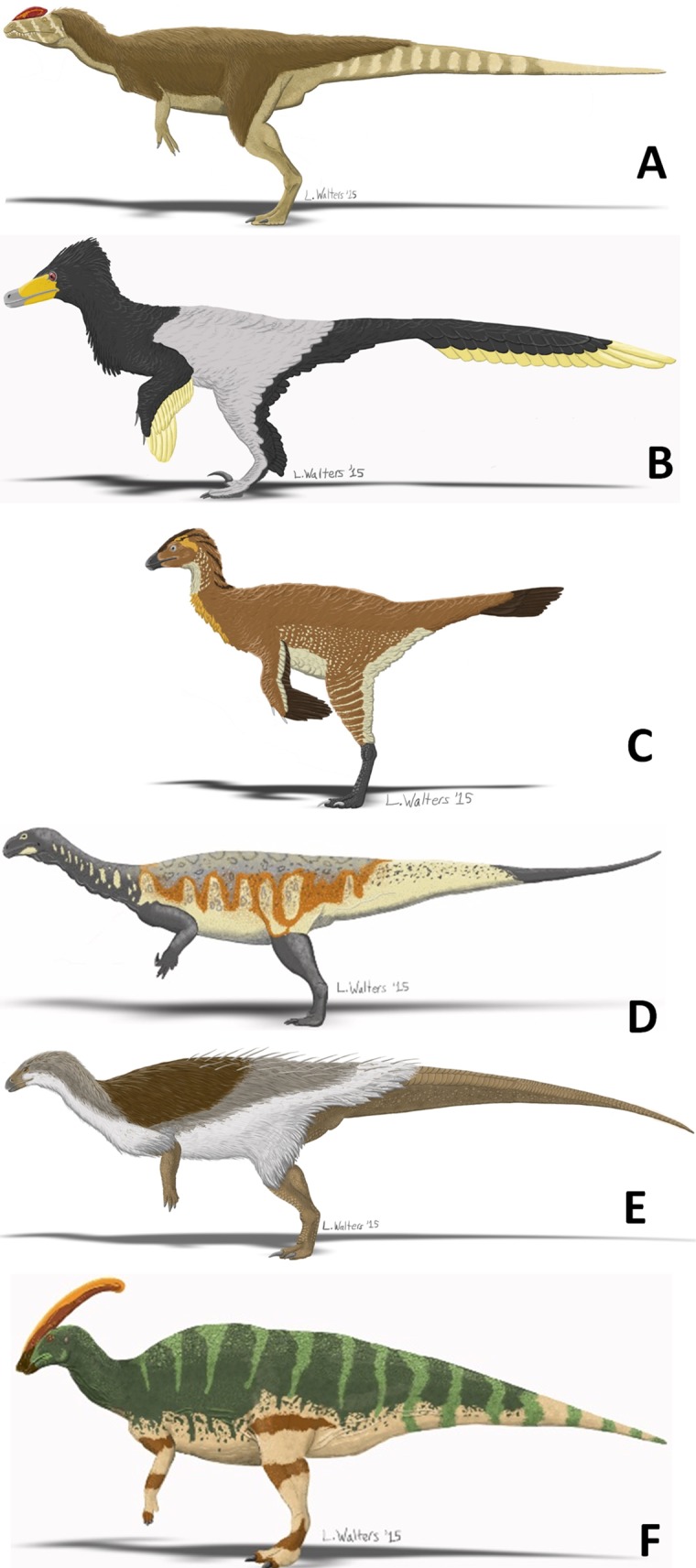
Reconstructions of dinosaurs from Fig 4 standing with the forelimb in resting pose, left lateral view. A. *Dilophosaurus wetherilli*. B. *Velociraptor mongoliensis*. C. *Caudipteryx* sp. D. *Plateosaurus engelhardti*. E. *Thescelosaurus neglectus*. H. *Parasaurolophus walkeri*. All images in this figure are reprinted from original, previously-unpublished artwork by Leandra Walters under a CC BY license, with permission from Leandra Walters, original copyright 2015.

Table 6 shows our recommended resting orientations at all joints for all taxa, based on these results. Fig 4 puts these recommendations into graphic form for the scapula and forelimb bones. Fig 5 puts these recommendations into graphic form for fleshed-out reconstructions.

## Discussion

In a plethora of saurischian skeletons preserved in articulation, the neck is arched backward over the dorsum. If such arching were accompanied by bodywide tetanic contractions, as has previously been suggested [72], then it would introduce artifacts into our results. This is because our study is based on the assumption that each specimen’s limb orientation is the product of elastic recoil during muscle relaxation and not the product of muscle contraction. However, a recent study showed that the arched-neck “death pose” of saurischians can be explained simply by immersion in water without muscle contraction [73]. This supports the idea that the angles measured here are the results of postmortem relaxation, not tetanic contractions, and are therefore unaffected by such artifacts. Also, it is very unlikely that such artifacts affect our ornithopod sample (including hadrosaurids), because in this sample all individuals exhibit a lack of flexion in enough limb joints to rule out the presence of bodywide tetanus.

Our sample sizes are small because of the scarcity of relatively complete dinosaurian fossils preserved lying on their sides in articulation. We therefore do not expect that our results will be the last word on scapular and forelimb orientations in dinosaurs. Our recommended scapular and forelimb orientations will probably change somewhat upon addition of future finds to the samples. However, despite small sample sizes, the available samples reveal important similarities and differences in scapular and forelimb orientation in different dinosaurian groups. An exception is Sauropodomorpha, for which scapulae and forelimbs preserved in articulation are particularly rare. Particular skepticism should be exercised concerning our recommendations for bony orientations in that group (Table 6), because our sample size is particularly small. Our recommendations for scapular and forelimb orientations in theropods and bipedal ornithischians are based on larger sample sizes and are therefore more reliable.

Bipedal saurischians exhibit the most horizontal scapulae among the dinosaurs we measured. The other groups we measured are all ornithischian taxa in which the vertebral column is arched strongly ventrally at the anterior end of the torso. The more horizontal scapular orientation of bipedal saurischians appears to be due to the lack of a strong arching of the vertebral column in the thoracic region. This orients the glenoid in such a way as to maximize the anterior reach of the forelimbs, which is important for an animal that uses its forelimbs in prey capture. Protraction of the humerus in carnivorous saurischians is limited [17–19,74,75], and they therefore benefit from having the glenoid oriented to maximize anterior humeral reach. This scapular position is facilitated by having a vertebral column that is not strongly arched ventrally in the thorax as it is in ornithopods.

The elbow and wrist are much more acutely angled in theropods with semilunate carpals than in other dinosaurian groups (Figs 4 and 5). The rounded articular surface of the semilunate carpal allows motion in a greater arc than is allowed by the flatter carpals of other dinosaurs [17]. Long, vaned feathers on the forelimbs are known only in theropods with semilunate carpals [7,30,33,58,76,77], whereas integumentary filaments on the forelimbs of other theropods are short and without broad vanes [78–80]. There thus appears to be a functional connection between ability to strongly flex the forelimb and possession of long, vaned feathers. This may be because intact feathers are conducive to display, gliding, and perhaps other functions, even in extinct theropods that did not use feathers for powered flight. The function of the strong flexing of the forelimb in feathered dinosaurs may be to avoid contact between feathers and the ground, so as to avoid damage to the feathers. This hypothesis is supported by the fact that extant birds avoid contact between feathers and the ground by keeping their forelimbs strongly flexed when at rest. It is also consistent with the exhibition of less resting forelimb flexion in Caudipteridae than in other feathered theropods, because caudipterid forelimbs are shorter than those of most other feathered dinosaurs [7,33,76], eliminating the possibility of contact between feathers and the ground, hence also eliminating the need for tight forelimb folding. This allowed the resting forelimb pose to approach the basal theropod condition in Caudipteridae.

It is noteworthy that the estimated resting pose of the elbow approximates maximum elbow flexion as found previously by range-of-motion studies in theropods with semilunate carpals [16,17,19], non-coelurosaurian theropods without semilunate carpals [17,18], and basal sauropodomorphs [20,81]. The estimated resting pose of the wrist also approximates maximum wrist flexion in theropods with semilunate carpals [16,17,19]. This suggests that, at these joints in these animals, the combined strength of the flexors is greater than that of the extensors, because these joints are drawn into flexion even when the only contractions occurring are those involved in muscle tone in resting muscles. These joints in these animals were therefore capable of more powerful flexion than extension. Powerful elbow and wrist flexion is important in an animal that uses its forelimbs to carry loads, because such flexion resists the pull of gravity. It is also important in a predator that uses its forelimbs in prey capture, because such flexion resists the attempts of prey to lunge away from the predator.

The resting shoulder angle in saurischians, as found here, is not close to the angle of maximum humeral retraction that was found in previous range-of-motion studies [17–21]. These animals therefore did not habitually carry their arms with the humerus in full retraction. Plausibly, avoidance of habitual humeral retractor contraction conserved metabolic energy.

It is also noteworthy that, for a given specimen, the left and right scapular blades often differ in inclination (Tables 1 and 2), even when the specimen is preserved still encased in sediment with integumentary impressions that suggest extended persistence of the skin after death (e.g. AMNH 5240, *Corythosaurus casuarius*, in which left and right scapular blade inclinations differ by 6°). This supports the hypothesis that dinosaurian scapulocoracoids were mobile [82], as in extant non-avian tetrapods [83–85]. Left and right scapulocoracoids of oviraptorosaurian and dromaeosaurid theropods are tightly coupled via the sternum [5,60], hence probably exhibited reduced mobility relative to each other, but such is not the case with other dinosaurian taxa. The recommended scapular blade orientations in Table 6 should therefore be treated not as immutable values but as values from which a reconstructor may safely stray a few degrees.

The most strongly vertical scapular blades are found in basal ornithopods (Fig 4). Their scapulae are approximately parallel to the anterior end of the dorsal vertebral series, which is strongly arched ventrally. The base of the scapular blade in hadrosauroid ornithopods is less vertical than in basal ornithopods, and a kink in the hadrosauroid scapular blade—absent in the blades of basal ornithopods—reorients the distal end of the blade so that it is nearly horizontal (Fig 4G). Due to the kink, hadrosauroids are the only dinosaurs in which the distal end of the scapula is further from the vertebral column than the acromion is. Range of motion studies on the forelimbs of hadrosauroids have not yet been published, so any paleobiological inferences made from hadrosauroid scapular and forelimb orientation must be treated with caution. However, if range of shoulder and elbow motion was similar between basal ornithopods and hadrosauroids, the more horizontal hadrosauroid scapular blade would have allowed greater anterior reach than in basal ornithopods, because maximal stretching of humeral extensors (which are attached to the scapular blade) during protraction would occur at a greater degree of humeral protraction than in dinosaurs without such a kinked scapular blade. This would have allowed food to be brought to the mouth by the hands more easily in hadrosauroids than in basal ornithopods.

The results of this study can also be applied to studies of dinosaur forelimb function, because accurate measurements of the limits of humeral motion through the transverse plane depend on accurate orientation of the glenoid, hence accurate orientation of the pectoral girdle. Theropod scapular blades were oriented at an angle near 21°, as recommended here for theropods, in some such studies [12,18,19,86]. However, scapulae were oriented differently in functional studies of the forelimbs of other theropods [16,17,75,87]. Humeral range of motion in cranial view may therefore have to be remeasured for the theropod taxa covered in those studies.
